# More Smoke

**DOI:** 10.4103/0973-1229.27617

**Published:** 2006

In the hot sultry fiasco of damp
enthusiasms
and silver rays in gloomy
archives,
narcosis and the feeling
of melting ice on frozen palms.

Arborescent shadows of sorrow
in reminiscent silhouettes
the rain soaked evening
of pouring emotions
and grassy long walks on dripping
pavements
Seeking out each other.

Remember, my dear
the sweet faint lavender
in the cleft of your breasts
hidden from the present and
posterity
and the knowledge of transience.

Looking at the grey skies
I think of clear blue water
and simmering passions
although
I've heard
you've created a smoke screen
from tall chimneys.
Ajai R. Singh

